# Hydroxychloroquine and QT Prolongation in Older Patients with Rheumatic Diseases: Who is afraid of the Boogeyman? We are not!

**DOI:** 10.31138/mjr.31.4.433

**Published:** 2020-12-22

**Authors:** Alberto Castagna, Giovanni Ruotolo, Ciro Manzo

**Affiliations:** 1 Azienda Sanitaria Provinciale Catanzaro, Geriatric Medicine department - Fragility Outpatient Clinic, Casa della Salute Chiaravalle Centrale, Catanzaro, Italy; 2 Azienda Ospedaliera Pugliese-Ciaccio di Catanzaro, Geriatric Unit, General Hospital, Catanzaro, Italy; 3 Azienda Sanitaria Locale Napoli 3 sud, Internal and Geriatric Medicine department – Geronthorheumatological Outpatient Clinic, poliambulatorio “Mariano Lauro”, Sant’Agnello, Naples, Italy

Dear Editors,

The role of hydroxychloroquine (HCQ) sulphate as a therapeutic option in coronavirus disease 2019 (COVID-19) patients aroused great interest and hope.^1–3^

In these months, the possibility that HCQ could cause adverse cardiac events when used in association with azithromycin has been brought to the attention of the scientific, and not only, community. In particular, Chorin et al*.* in patients treated with HCQ and azithromycin, observed prolongation of the corrected QT interval (QTc) from a baseline average of 435 ± 24 ms (mean ± sd) to a maximal average value of 463 ± 32 ms (*P*< 0.001 (one-sample *t*-test)) which occurred on day 3.6 ± 1.6 of therapy.^4^ Mercuro et al. observed that patients who received HCQ for the treatment of pneumonia associated with COVID-19 were at high risk of QTc prolongation, and concurrent treatment with azithromycin was associated with greater changes in QTc.^5^

As known, QT is the time from the start of the Q wave to the end of the T wave. It represents the time taken for ventricular depolarisation and repolarisation, effectively the period of ventricular systole from ventricular isovolumetric contraction to isovolumetric relaxation. The QTc estimates the QT interval at a standard heart rate of 60 bpm. This allows comparison of QT values over time at different heart rates and improves detection of patients at increased risk of arrhythmias. The normal QTc interval in adults is 0.36 to 0.47 seconds (360–470 milliseconds) in males and 0.36 to 0.48 seconds (360–480 ms) in females. In a small percentage of patients, QT interval prolongation (defined as ≥ 470 ms in males and ≥ 480 ms in females), can trigger torsades de pointes (TdP). This risk is not a linear function of QTc values. TdP is, by definition, a form of polymorphic ventricular tachycardia with a heart rate greater than 100 beats per minute with characteristic twisting around the isoelectric baseline every 5–20 beats. TdP is associate with a high risk of sudden death.^6^

Among the known medications reported to cause QT interval prolongation, HCQ is not commonly implicated. In 2017, during the Malaria policy advisory committee meeting organised by the World Health Organization (WHO), no case of arrhythmic death was reported.^7^ In 2018, a systematic review article reported that the risk of cardiac adverse events (conduction disorders, among these) was not quantifiable because of the lack of randomised controlled trials and observational studies investigating this association.^8^ To date, only case reports and case series of very small size are present in the literature.

The proposed mechanism by which HCQ causes QT interval prolongation is not well understood. Capel et al. observed in guinea pig sinoatrial node myocytes findings consistent with inhibitory effects of HCQ on the hyperpolarization activated current ion channels (also known as “funny current” channels), along with delayed rectifier potassium currents and L-type calcium ion currents. These inhibitory effects on the pacemaker cells were shown to cause delayed rates in depolarization leading to decreased heart rates.^9^

It is a common knowledge that QTc increases with age, and recent data suggest that frailty-related phenotypes are associated with QTc prolongation. For instance, inactivity and light-intensity physical activity were associated with QTc prolongation in older adults.^10^

With the progressive lengthening of life, the possibility that inflammatory rheumatic diseases are diagnosed in the elderly is increasing. In older patients, polypharmacy is frequent, and age-induced changes in pharmacokinetics may create completely different scenarios compared to adult or young populations.^1^

In clinical practice, the risk for QTc interval prolongation must always be assessed in older patients affected by inflammatory rheumatic diseases, before starting HCQ therapy. Particular caution is advised when combining QT-prolonging medications (**Table 1**).

**Table 1.** Drugs associated with a known risk of QT prolongation.

**Drug Class**	**Known risk of QT prolongation**
Anaesthetic, general	Propofol, Sevoflurane
Antiarrhythmic	Amiodarone, Disopyramide Phosphate, Flecainide, Ibutilide, Procainamide, Quinidine, Sotatol
Antidepressant	Citalopram, Escitalopram
Anticancer	Arsenic Trioxide, Eribulin, Vandetanib
Antiemetic	Ondansetron, Droperidol
Antifungal	Fluconazole, Pentamidine
Antimalaric	Chloroquine, Halofantrine
Antipsychotic	Chlorpromazine, Haloperidol, Pimozide, Thioridazine
Antibiotic	Azithromycin, Clarithromycin, Erythromycin, Ciprofloxacin, Levofloxacin, Moxifloxacin
Cholinesterase inhibitor	Donepezil
Illicit Drug	Cocaine
Opiates	Methadone
Phosphodiesterase 3 Inhibitor	Anagrelide, Cilostazol

In 2013, Tisdale et al. proposed an algorithm to quantify this risk.^11^ Maximum score is 21, and total score classified patients in high (equal or higher than 11 points), moderate (7–10 points) and low risk (less than 6 points) (**Table 2**). In our clinical practice, routine use of the Tisdale scale proved very useful to identify and minimise this specific risk.

**Table 2.** Calculation of risk score for QTc interval prolongation (modified by Tisdale et al., 2013).

**Risk Factor**	**Point**
Age≥68 years	1
Female Sex	1
Loop Diuretic	1
Serum Potassium ≤3,5 mEq/L	2
Admission QTc ≥450 ms	2
Acute Myocardial Infarction	2
2 QTc Prolonging Drugs*	3
Sepsis	3
Hearth Failure	3
One QTc Prolonging Drugs*	3
**Maximum Risk Score**	**21**

* Three points for taking one QTc interval–prolonging drug; 3 additional points for taking ≥2 QTc interval–prolonging drugs (for a total of 6 points).

In line with Tisdale’s algorithm, an electrocardiographic (ECG) control is fundamental as well as it is prudent to correct electrolyte disorders (mainly hypokalaemia and hypomagnesaemia), and, where possible, avoid or minimize use of other drugs known to prolong the QT interval in relation to comorbidity and poly-pharmacotherapy. History of long QT syndrome, or baseline QTc prolongation, or Tisdale risk score > 11 should suggest other different drugs or, alternatively, a tight and short-time ECG control.

Moreover, as already highlighted, randomised controlled trials and observational studies investigating the association between HCQ and QTc values are still lacking. Routine use of the Tisdale scale could provide useful information regard this potential risk.
